# Effect of WC Content on Microstructure and Properties of CoCrFeNi HEA Composite Coating on 316L Surface via Laser Cladding

**DOI:** 10.3390/ma16072706

**Published:** 2023-03-28

**Authors:** Jiang Huang, Zhikai Zhu, Hao Wang, Kaiyue Li, Wenqing Shi, Tianwen Jiao

**Affiliations:** 1 School of Electronics and Information Engineering, Guangdong Ocean University, Zhanjiang 524088, China; 2 Guangzhou Shipyard International Company Limited, Guangzhou 511462, China

**Keywords:** overlapping rate, laser cladding, HEA, hardness, corrosion resistance

## Abstract

Laser cladding technology is used to fabricate CoCrFeNi HEA/WC composite coatings with different mass fractions of WC on the surface of 316L stainless steel. The microstructures of HEA/WC composite coatings were analyzed by combining multiple characterization techniques. The results show that the HEA/WC composite coatings have good surface formation without pores and hot cracks, and the metallurgical bonding is well formed between the coating and the 316L SS substrate. Under the action of a laser beam and molten pool, WC particles partially or slightly melt and diffuse to the matrix, which hinders the orderly growth of grains and forms multiple strengthening. The phase structure of the HEA/WC composite coatings is composed of a main phase with FCC. The hardness and corrosion resistance of the HEA/WC composite coatings are clearly enhanced, and the HEA/WC composite coating with 5% WC has optimum properties.

## 1. Introduction

The surface is the most easily damaged part of a material. In order to improve the surface properties, various surface coating technologies have been developed [1,2,3,4,5]. Among them, laser cladding technology is considered one of the most promising surface technologies due to its advantages such as no pollution, low price, low dilution rate, wide application range and so on. At present, laser cladding technology has become one of the important methods for preparing new materials, repairing and remanufacturing failed metal parts, and it has been widely used in aviation, machinery manufacturing, petrochemical industry, shipbuilding and other industries.

High-entropy alloys (HEA) are a new type of alloy developed in the last twenty years that generally contain more than five principal components [6,7,8,9,10]. HEA have some unique advantages, such as strong corrosion resistance, high hardness, high temperature strength and high toughness [11,12,13], which have attracted attention in industry, energy fields, aviation and academia [14]. At present, due to the limitation of production level, HEA are mainly powders and thin slices, which cannot satisfy the requirements of industrial application. Laser cladding technology can prepare HEA powder for use as a coating on the surface of metal materials, so that the metal materials present the specific properties of HEA. CoCrFeNi HEA is a new type of single-phase solid solution HEA with excellent mechanical properties that has attracted wide research interest [15,16,17]. Many kinds of ceramic reinforcing particles have been investigated. Guo Y X et al. [18] found that the wear resistance and hardness of CoCrFeNi HEA were improved by adding in situ TiN reinforcement particles. Shu et al. [19] and Zhang et al. [20] found that the proportions of Co, Cr, Fe and Ni elements also have an important influence on the properties of the CoCrFeNi HEA coating. Recently, Han B et al. [21] compared high-speed laser cladding of CoCrFeNi HEA with traditional laser cladding. They found that for the coating obtained by high-speed laser cladding, the phase structure is a single face-centered cubic (FCC) solid solution, the grain size is finer and the hardness is higher. Although the hardness obtained by high-speed laser cladding is higher, it is only about 380 HV. Improving the comprehensive properties of CoCrFeNi HEA requires further study. As it is known that WC ceramic particles possesses high hardness as well as thermal and chemical stability, WC reinforcement particles are already applied in many coating systems [22,23,24,25]. However, there are few reports about adding WC particles to CoCrFeNi HEA.

316L SS is a typical austenitic stainless steel that is not only cheap to manufacture, but also rich in raw materials, and it is widely used in nuclear power plants, ships and bridges. In this study, CoCrFeNi HEA composite coating with 0%, 2.5%, 5.0%, 7.5% and 10.0% mass fraction of WC were fabricated for 316L SS using traditional laser cladding technology. The microstructure characteristics and mechanical properties of the HEA/WC composite coatings were analyzed by combining multiple characterization techniques.

## 2. Experimental Processes

### 2.1. Material and Coating Preparation

**Figure 1:**
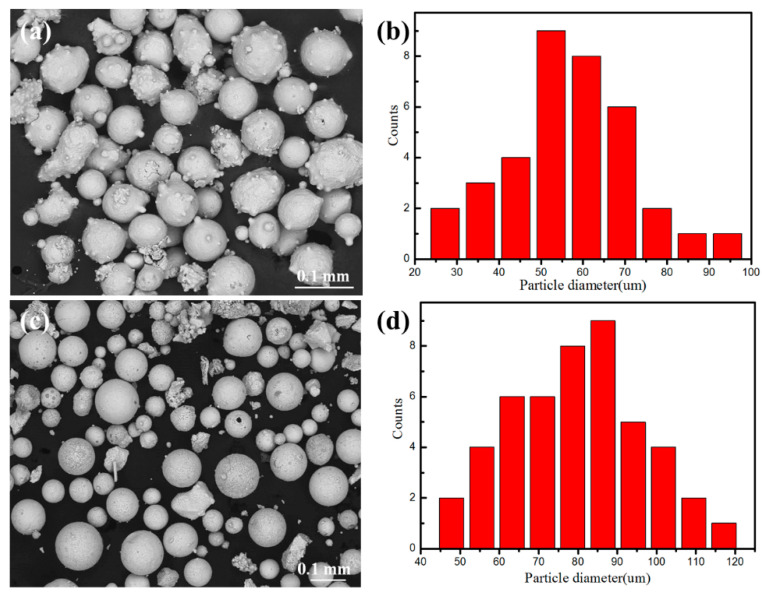
SEM morphology: (**a**) CoCrFeNi HEA powders; (**b**) spherical WC powders; (**c**) the distribution of CoCrFeNi HEA powders; (**d**) the distribution of spherical WC powders.

The 316L SS steel with dimensions of 100 mm (length) × 50 mm (width) × 2 mm (thickness) was prepared as substrate. The surface of the 316L SS was ground with abrasive papers and decontaminated with ethanol before the experiment. The microscopic morphologies and particle size analysis of the CoCrFeNi HEA powders and the spherical WC particles are shown in Figure 1, and Table 1 shows the chemical composition of 316L SS. The spherical WC particles and CoCrFeNi HEA powders were mixed using a CZ0001 planetary ball mill for 2 h and dried in an oven for 2 h at 100 °C. The marking methods of CoCrFeNi HEA coatings with different mass fractions of WC particles are shown in Table 2. The multi-track coatings were fabricated using the XL-F2000W fiber continuous laser processing system (model: XL-F2000 W, manufacturer: Maxphotonics Co., Ltd., Shenzhen, China) with a maximum output power of 2 kW, a circular Gaussian beam and a spot diameter of 2.5 mm. The wavelength of the laser beam was 1080 nm, the experiments used preset powder mode, the laser scanning speed was 700 mm/min with a 50% overlapping rate and the defocus was +2 mm on the basis of our previous work experiences and some similar research works. The powder spreading thickness was 1 mm by using a standard mold.

### 2.2. Test Methods

The HEA/WC coatings were cut with a size of 10 mm (length) × 10 mm (width) × 2 mm (thickness) using a wire cutting machine in order to analyze their microstructure. The microstructure was analyzed using a scanning electron microscope (SEM, FEI, Quanta 250 FEG, Hillsboro, OR, USA) and an optical microscope (OM, XJL-302/302BD, Yuexian Optical Instruument Co., Ltd., Guangzhou, China). The chemical compositions of the samples were determined with an energy dispersive spectrometer (EDS). An X-ray diffractometer (XRD, XRD-6100, Shimadzu, Kyoto, Japan) was used to investigate the characteristics of the phase constitutions of the composite laser cladding coatings with a scanning speed of 7°/min. According to the actual situation, the scanning angle of was 20° to 80° selected. An electrochemical workstation (CHI660E, Chen Hua Instruments, Shanghai, China) was used to measure the samples’ corrosion resistance in a 3.5% NaCl solution at room temperature. The scanning range of test voltages was −1.4 V to +0.5 V at room temperature.

## 3. Results and Discussion

### 3.1. Morphology Analysis of HEA Composite Coatings

**Figure 2:**
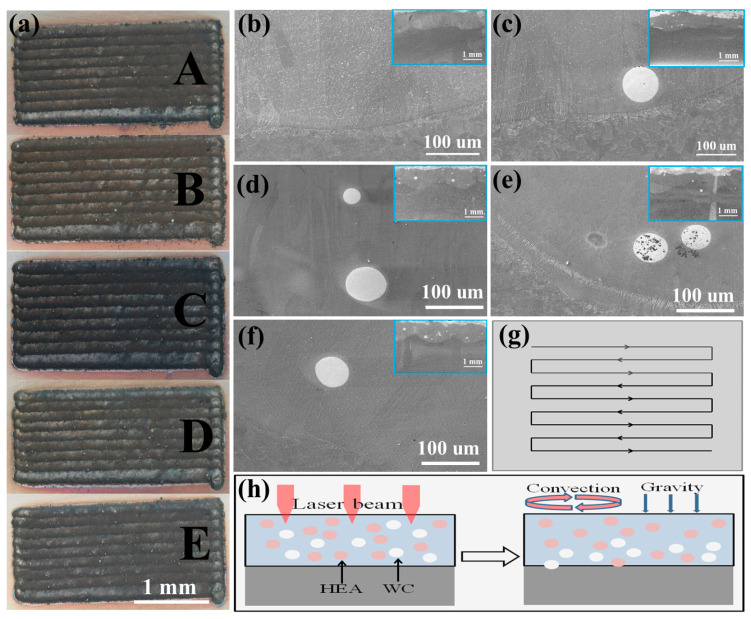
Morphology of HEA/WC coating. (**a**) HEA/WC coating surface; (**b**) A coating section; (**c**) B coating section; (**d**) C coating section; (**e**) D coating section; (**f**) E coating section; (**g**) scanning path diagram; (**h**) convection and gravity model of HEA/WC coating during solidification.

Figure 2 shows the surface and cross-sectional morphology of the laser cladding HEA/WC composite coatings. In Figure 2a, the surfaces of the cladding coatings are well formed on the 316L SS substrate without obvious pores or hot cracks. Figure 2b–f shows the cross-sectional morphology of the laser cladding samples A, B, C, D and E, respectively. It can be seen that the coating and the substrate are well connected, and the interfaces are flat and smooth with no pores or hot cracks. The metallurgical bonding is well formed. Figure 2g is the scanning path diagram of multi-track laser cladding. The morphology of the WC particles keeps its original spherical shape, and only a very small number of them are decomposed and melted, such as in Figure 2e. Figure 2h is a model of the laser interaction and coating solidification process. On the one hand, due to the influence of the convection effect, the WC particles and CoCrFeNi HEA powder melt to each other uniformly in the high-temperature molten pool. On the other hand, the density of WC particles is much higher than that of CoCrFeNi HEA powder, so most WC particles move to the bottom of the coating under the influence of gravity [26,27]. 

### 3.2. Microstructure and EDS Mapping Analysis

**Figure 3:**
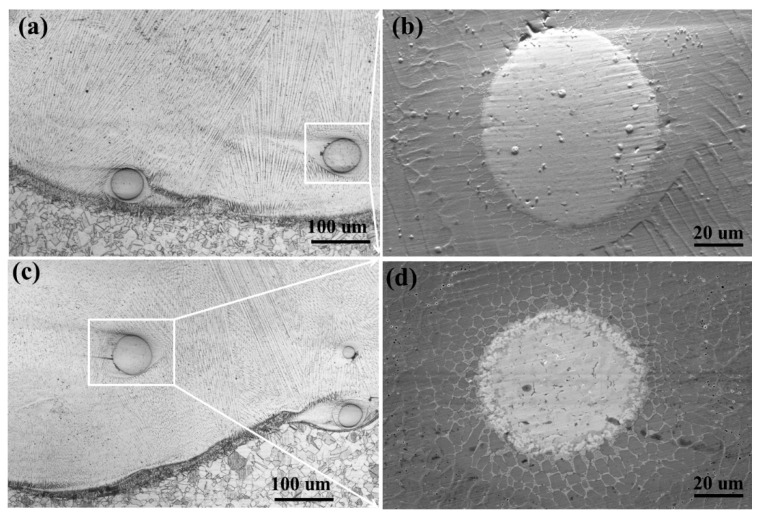
Dendritic morphology of coatings with different compositions (**a**) B coating section with WC particle; (**b**) magnification of B coating section; (**c**) E coating section with WC particle; (**d**) magnification of E coating section.

Figure 3 shows the cross-sectional dendrite morphology of the microstructure of the HEA/WC composite coating. Laser cladding is a typical non-equilibrium solidification process; the solidification structure is determined by the ratio of the temperature gradient (*G*) and the solidification rate (*R*) due to the constitutional undercooling criterion [28,29]. Under the action of the laser beam, the substrate surface melts with the HEA/WC composite powder, and solidifies with heat dissipation. In the solid–liquid interface, the crystal specific value *G/R* is very large. As a result, the growth rate is much slower than the nucleation rate, the crystal grows along the direction of heat dissipation and the planar crystal can be generated, as shown in Figure 3a,c. In the region without WC particles, the columnar crystals in the coating grow vertically to the interface. In the region around the WC particles, the columnar crystals that grow directionally in the coating become fine cellular crystals and the growth direction becomes disordered, as shown in Figure 3b,d. The WC particles play a role in hindering the growth of columnar crystals. There are only a few WC particles melted, damaged and decomposed in the coating. The effects of adding WC particles are as follows: (1) the crystal size is refined around the WC particles and (2) the WC particles hinder the growth of columnar crystal so an unordered cellular crystal is obtained.

**Figure 4:**
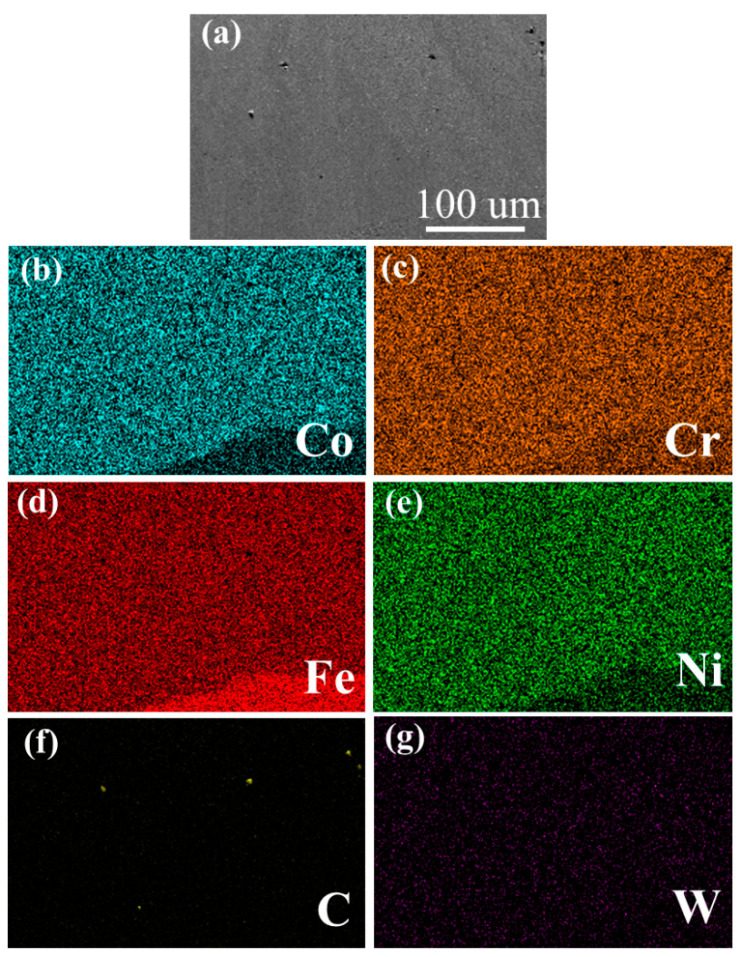
EDS mapping of CoCrFeNi HEA coating of sample A. (**a**) SEM of the CoCrFeNi HEA coating; (**b**) Co element distribution; (**c**) Cr element distribution; (**d**) Fe element distribution; (**e**) Ni element distribution; (**f**) C element distribution; (**g**) W element distribution.

Figure 4 shows the EDS mapping test results of the coating of sample A. It can be seen that the Co, Cr, Fe and Ni elements are evenly distributed in the coating; there is no component segregation. The Co/Fe elements form a clear dividing line on the interface, while there is almost no change in the distribution of the Cr/Ni elements. Combined with the information displayed in Table 1, the elements in the composite coating penetrated into the substrate, indicating that their combination was good, and the dilution degree is small. There is nearly no W/C element in the whole coating.

**Figure 5:**
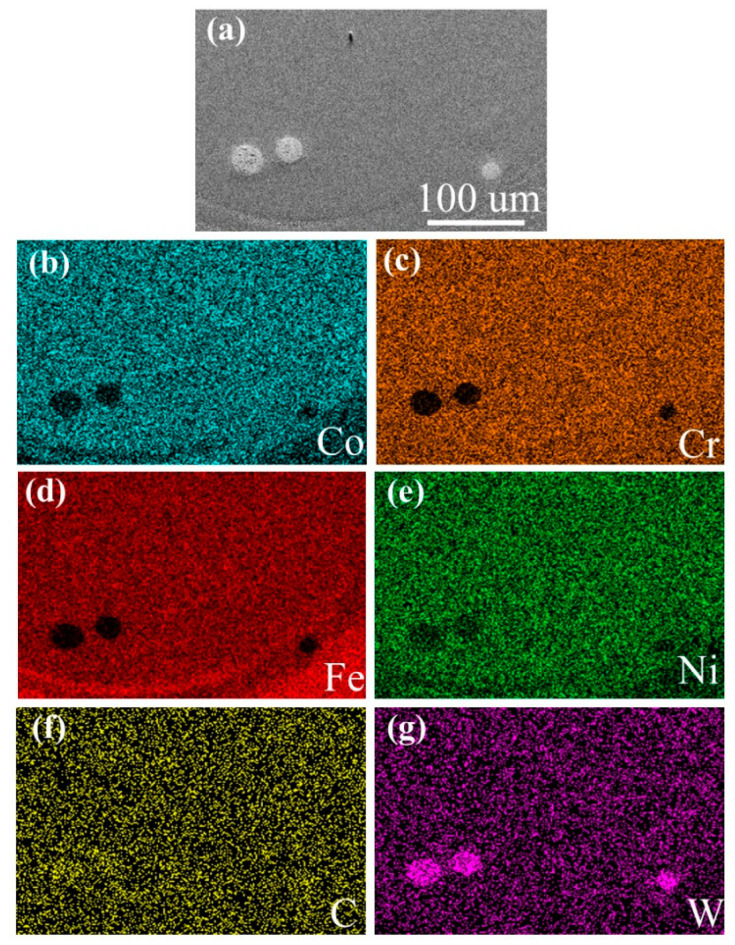
EDS mapping of HEA/WC composite coating of sample D around the WC particles. (**a**) SEM of the HEA/WC composite coating; (**b**) Co element distribution; (**c**) Cr element distribution; (**d**) Fe element distribution; (**e**) Ni element distribution; (**f**) C element distribution; (**g**) W element distribution.

In order to analyze the microstructure of the coating around the WC particle, the coating of sample D was selected for the EDS mapping, and the EDS mapping test results are shown in Figure 5. It can be seen that a spherical WC particle appears in the coating, and a certain amount of W/C elements are distributed around the WC particles, which indicates that some WC particles decompose under the synergistic effect of the high-temperature molten pool and high-energy laser beam. The decomposed WC enhances the content of W/C elements in the composite coating. W/C elements combine with other elements to form carbide. Carbide increases the nucleation rate, which makes the grain refined, resulting in grain refinement strengthening, and improves the hardness of the coating [30]. 

**Figure 6:**
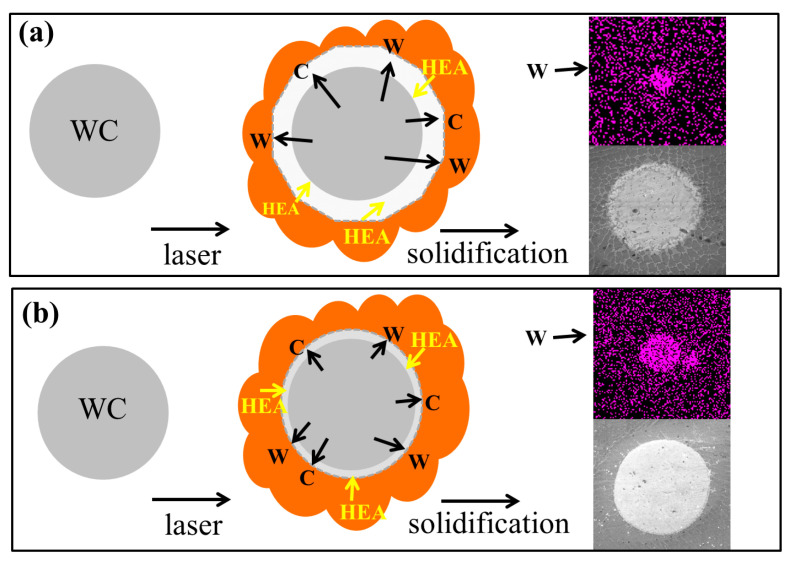
Schematic diagram of decomposition and solidification of WC particles. (**a**) Partial decomposition; (**b**) slight decomposition.

Xiao Q I et al. [31] divided the melting and decomposition of WC particles into three categories. According to the above analysis, it can be concluded that the situation in this experiment belongs to partial decomposition and slight decomposition. Figure 6a shows the schematic diagram of the decomposition and solidification of a partially decomposed WC particle. Under the interaction of the high-energy laser beam and high-temperature molten pool, the edge of incompact WC particles can be partially decomposed, then the melted part of the WC particles are injected into the molten pool. Under the action of the convection effect, as shown in Figure 2h, the decomposed WC particles diffuse into the molten pool to form new carbides. These carbides enter the microstructure, promoting the solid solution strengthening. In addition, the nucleation starts from the interface coating substrate, and spherical WC particles stop the growth of HEA grains, thus forming disordered refined grains. Figure 6b shows the slight decomposition and solidification mechanism of compact WC particles. The WC particles remain spherical, and the thermal action of the molten pool and laser beam is the physical mechanism of WC particle decomposition and solidification.

### 3.3. Phase Composition 

**Figure 7:**
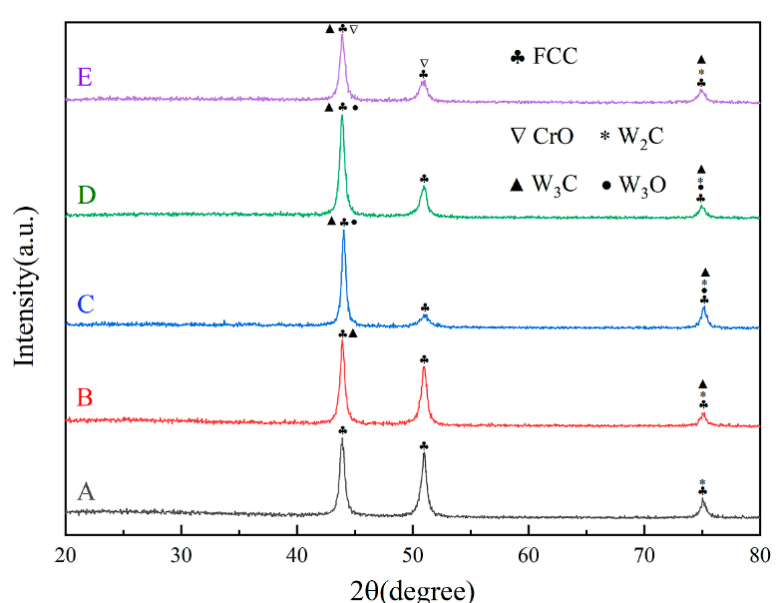
XRD pattern of HEA/WC laser cladding coating.

Figure 7 shows the phase composition of the CoCrFeNi HEA composite coatings fabricated using laser cladding. It can be seen that sample A only exhibits a single FCC phase (JCPDS: no. 33-0397; no. 33-0945; no. 34-0396), which is consistent with other research [32,33]. With the addition of WC particles, some carbides and oxides, such as W_2_C (JCPDS: no. 20-1315), W_3_C (JCPDS: no. 42-0853), W_3_O (JCPDS: no. 41-1230) and CrO (JCPDS: no. 08-0254), gradually appeared in the diffraction peak, which reveals that the WC particles melt and decompose. The formation of oxides may be based on the air mixed into the powders. However, there is no diffraction peak of WC in the XRD pattern, which may be due to the low content of WC, and some of it is decomposed and burned during laser cladding [34].

### 3.4. Properties of the HEA/WC Composite Coating

**Figure 8:**
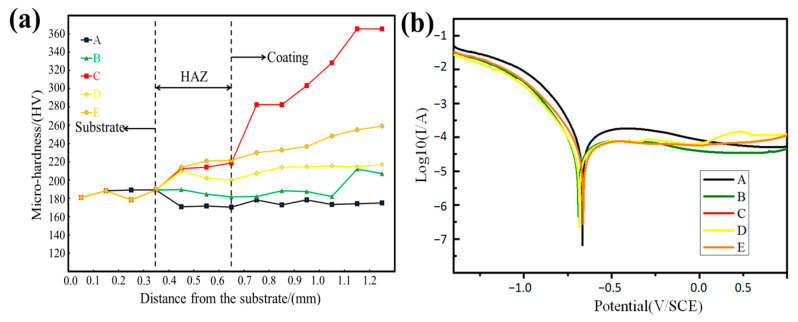
Mechanical properties of the HEA/WC coating. (**a**) The hardness distributions of HEA/WC composite coatings; (**b**) the potentiodynamic polarization evolution curves of HEA/WC composite coatings.

Figure 8a shows the hardness distribution of the cross section. The hardness of the HEA/WC composite coating is roughly divided into three levels. Firstly, the hardness of the substrate is about 185 HV, which is because the substrate is 316L SS. Secondly, in the heat affect zone (HAZ), the hardness of samples B, C, D and E are increased, which was not the case for sample A. It is well known that the HAZ is around the interface of the coating and substrate; the high-hardness WC particles diffuse to the interface due to the convection effect, thus improving the hardness. While sample A is pure CoCrFeNi HEA, its hardness is low, about 170 HV [35]. Thirdly, it can be seen that sample C has the highest hardness, though the hardness of samples B, D and E are also enhanced in the coating. This is mainly caused by the multiple strengthening actions of refined grain strengthening and solid solution strengthening effects [30]. 

We studied the corrosion characteristics at room temperature and used three-electrode cells: a reference electrode, a working electrode and an auxiliary electrode. The experiment was repeated at least three times to ensure reproducibility. Test samples were made according to the standard of 1 mm × 1 mm. The electrochemical performance of the sample was tested after exposure to 3.5% NaCl for 30 min. The Tafel curves of the samples A, B, C, D, E and 316L SS are shown in Figure 8b, using the Tafel slope extrapolation method [36] in Table 3. Compared with the 316L SS substrate, the HEA/WC coatings have a higher corrosion potential, and sample C has the highest corrosion potential. According to the corrosion criterion, the higher the corrosion potential, the stronger the corrosion resistance [37]. Therefore, it can be proven that the corrosion resistance of the HEA/WC coatings has been improved. WC has good corrosion resistance. Adding a small amount of WC can improve the corrosion resistance of a coating. However, if the mass fraction of WC is too high, the WC will decompose under the heat of the laser beam to generate some compounds and oxides, thus reducing the corrosion resistance. However, the corrosion current density has an irregular evolution, so it needs further study.

## 4. Conclusions

HEA/WC composite defect-free coatings with different components were fabricated on the surface of 316L SS. WC particles can hinder the growth of directional dendrites and promote the growth of disordered cellular grains. 

WC particles show partial decomposition and slight decomposition in the composite coatings under the interaction of a laser beam and molten pool. The decomposed WC diffuses to the molten pool to form hard carbide, which plays a role in solid solution strengthening. Meanwhile, spherical WC particles stop the growth of HEA grains, thus forming disordered refined grains.

The hardness of HEA/WC composite coatings is clearly enhanced by multiple strengthening actions, and the coating with 5% WC has the highest hardness. The electrochemical experiments show that the corrosion potential of HEA/WC composite coatings is improved, and the coating with 5% WC has the highest corrosion potential. 

## Figures and Tables

**Table 1:** Chemical composition of 316L SS (mass fraction, %).

Cr	Ni	Mn	Mo	Si	Fe
16–18	10–14	2	2–3	1.5	Bal.

**Table 2:** Coating numbers with different WC mass fractions.

Number of Coating	Content of WC/(Mass.%)
A	CoCrFeNi HEA + 0%WC
B	CoCrFeNi HEA + 2.5%WC
C	CoCrFeNi HEA + 5.0%WC
D	CoCrFeNi HEA + 7.5%WC
E	CoCrFeNi HEA + 10%WC

**Table 3:** The samples’ E_corr_ and I_corr_.

Materials	E_corr_ (V/SCE)	I_corr_ (A/cm^2^)
A	−0.666	6.549 × 10^−8^
B	−0.689	4.988 × 10^−7^
C	−0.660	2.376 × 10^−7^
D	−0.684	2.222 × 10^−7^
E	−0.662	6.985 × 10^−7^
316L SS	−0.705 [38]	8.184 × 10^−7^ [38]

## Data Availability

Not applicable.
